# Cure for the Ring-Worm

**Published:** 1812-03

**Authors:** 


*To the Editors of the Medical and Physical Journal.*

GENTLEMEN,

ON looking over some of your late Journals this evening, I met with the inquiry of Philanthropos (in the Journal for July of last year) for a remedy for "ring-worm." This complaint has been, during the last year, particularly extensive and troublesome, so much so as to baffle our "cutaneous doctors" of deserved eminence. It is only within a fortnight that I have been acquainted with an application which I have reason to believe is as deserving of the appellation "specific" as mercury is for the cure of syphilis.

I can detail several cases in which it effected a perfect and speedy cure, but it will be better to give you at once the secret; and I hope that neither you nor your readers will think the worse of it, when I tell you I had it accidentally from a lady, whose son was last month under my care for a remitting fever, and at the same time under her's for ring-worm. Both of us were successful: I saw our patient to-day free from disease.

Roll a sheet of writing-paper into a cylinder about half an inch in diameter; set fire to one end of it by holding it to the flame of a lighted candle or taper; apply the other, through which the smoke issues, to the ring-worm. In this manner apply the smoke of the burning paper to the ring-worm for fifteen minutes at a time, once or twice a-day, as long as any appearance of disease remains. It seems a very few "smokings" in this manner will effect a cure. If the

 hair hair is thick, or perhaps it will in all cases be proper to shave the part diseased, and to rub off the scurf with a towel, before the first application of the smoke. If properly done, the skin soon acquires a deep yellow tinge.

I am concerned to find that a "Norfolk Practitioner," in your Journal for November, questions the veracity of one of your frequent correspondents, Mr. Knowles; whose reply, in your last number, I conceive is not quite satisfactory in rebutting the charge. Mr. K. should have stated the name of the person, and the borough-town in North Wales, where he states the case of "ascites" happened. This would have been a sufficient answer to any anonymous charges. The N. P. can avail himself of the challenge to communicate by letter with Mr. K. for further information, but probably the reasons which induced the N. P. to conceal in the first instance his name, will prevent his now disclosing it.

It is a most unwarrantable attack on the character of Mr. K. if the N. P. adduced the charge on insufficient grounds. To accuse, and substantiate the accusation, that a man is guilty of an intentional and studied deviation from the truth, is to debase and sink him in the estimation of every honorable and consistent person, and to level him with those with whom intercourse is disgraceful. I hope Mr. K. will clear himself honorably; but I wish to make a remark or two on his reply, which, with his former papers, does not convince me that he has any extraordinary sagacity either in the investigation or treatment of disease.

His reply in a great measure consists of interrogatories, some of which I will not pretend to meddle with; but, when he asks the N. P. "whether he has ever seen mercury given in diarrhœa?" I really think he cannot be serious! Surely Mr. K. need not be told that a disturbed state of the biliary secretion is a very common exciting cause of diarrhœa, and that it is of the first importance to inquire into and investigate the nature of alvine evacuations? I conceive also that the N. P. had no idea of exciting salivation when he recommended "small alterative doses of mercury," as Mr. K. conjectures. Nor will mercury, administered with opium or rhubarb, or both, under such circumstances, augment the already too-great excitement of the body. Quite contrary I conceive will be its effects: it will allay irritation, by restoring the disordered secretion of the abdominal viscera to health. I wish to be understood as speaking now only of that kind of diarrhœa which can evidently be traced to a disordered state of the liver. But, again, Mr. K. asks, "If in the case of diarrhœa there had been any derangement in the functions of the alimentary canal, must it not have been productive productive of some of those symptoms which characterise dyspepsia, or some other affection of the stomach, which certainly was not the case: or, if irregularity in the secreting hepatic organs, obstructions in any of the ducts, preventing the bile from passing into the intestines *for the assistance of digestion!* would it not have been absorbed into the circulating fluids, and produce symptomatic affection, termed icterus, instead of anasarca? This is too long an interrogation to be considered collectively. It may appear very lucid to the ingenious writer; but it appears in a very different light to me. I will answer part of it in the author's method, first asking him whether diarrhœa does not consist in a deranged state of the functions of the alimentary canal? And does the bile pass into the duodenum for the purpose of "assisting in digestion?" I thought, till now, that the stomach performed that office without the assistance of the bile, which I have been taught to consider as secreted for a very different purpose. I will conclude by one more remark only, and that is, that I cannot agree with Mr. K. in the statement, that good intentions are always undeserving of criticism. I am, Gentlemen,

Your obedient and humble Servant,

An ESSEX PRACTITIONER.

*Jan.* 3, 1812.

P.S. I have lately purchased the 7th edit. of the London Practice of Physic, by E. G. Clarke, M.D. I wished the other day for information respecting dyspepsia; and, on examining the work with this view, I was surprised that no such disease, if my eyes have not deceived me, is there treated of. This is a serious omission in a work professing to be as perfect as possible, and so extensively circulated.

